# WHF Position Statement for United Nations Fourth High-Level Meeting-2025

**DOI:** 10.5334/gh.1467

**Published:** 2025-09-19

**Authors:** K. Srinath Reddy, Bente Mikkelsen, George A. Mensah, Philip J. Landrigan, Amam Mbakwem, Renu Garg, Jeremiah Mwangi, Sean Taylor, Pablo Perel, Borjana Pervan, Finn-Jarle Rode, Daniel Pineiro, Dorairaj Prabhakaran, Jagat Narula, on behalf of the World Heart Federation

**Affiliations:** 1 Public Health Foundation of India, India; 2 World Heart Federation, Switzerland; 3 Center for Translation Research and Implementation Science, National Heart, Lung, and Blood Institute, United States; 4 Global Observatory on Planetary Health, Boston College, United States; 5 Centre Scientifique de Monaco, Monaco; 6 University of Lagos College of Medicine, Lagos, Nigeria; 7 Resolve to Save Lives, New York, United States; 8 Department of Non-Communicable Disease Epidemiology, London School of Hygiene and Tropical Medicine, United Kingdom; 9 Department of Medicine, University of Buenos Aires, Argentina; 10 Centre for Chronic Disease Control, New Delhi, India

**Keywords:** Cardiovascular diseases, low-middle income countries, Sustainable development goals

## Assessing the Present to Alter the Future

As the world gauges progress towards the SDG targets set for 2030, it is becoming evident that most countries are not on track to achieving them. Collective resolve among all nations and pooled global resources are needed to accelerate progress to reach as close to those targets as possible. It is also clear that commitment to those targets must continue beyond 2030 since many low- and middle-income countries (LMIC) will most likely experience rising burdens of non-communicable diseases (NCD) for some decades beyond the SDG dateline and to ensure commitment from the global community. This is especially true for target 3.4, including cardiovascular diseases, cancer, diabetes, and chronic respiratory diseases, and mental health, which are responsible for over 43 million deaths worldwide every year, with 18 million dying prematurely before the age of 70 years, and also cause the majority of morbidity and disability.^1^ This is because ongoing demographic, nutritional, and environmental transitions in those countries will result in an accelerated incidence of NCD in the future. The inequities between and within countries are huge. The probability of premature deaths in Western Europe and Canada is as low as 15 percent, while it remains as high as 52 percent in Sub-Saharan Africa.

Over half of the world’s population lacks access to basic health services,^2^ and many low-income countries lack the financial resources necessary to elevate their healthcare resources to an acceptable level. Strengthening health systems is, therefore, crucial for reducing global health inequality. The urgency of action must be emphasized. While the rising cost of healthcare services will likely overwhelm social systems and public budgets, various critical measures needed to support control of NCD, including mental health, are beyond the scope of the health sector responsibilities. Managing the impending NCD crisis requires a more comprehensive effort that includes national health structures, economic development, trade and technology, finance and treasury, education, and social affairs. A concerted governmental approach is mandatory to implement proven interventions to save lives through sustainable health systems and economic policies.

## Redefining the Global NCD Target

As we rededicate our efforts to counter the threat posed by NCD to global health and development, there is also a need to revise the target set in SDG 3.4. Restricting the target to mortality between the ages of 30 and 70 years precludes attention to NCD affecting persons at ages below 30 years and over 70 years. These burdens are substantial even now and are likely to rise in future as metabolic and environmental degradation are disrupting health across the life course. People at all ages, if assisted with timely diagnosis and management of NCD, can lead socially productive and fulfilling lives. They need to be supported by prevention against the 5 main risk factors: tobacco, alcohol, physical inactivity, unhealthy food and air-pollution and to have access to efficient health and social systems. Although children with NCD were never part of the SDG agenda beyond vaccination and maternal and child health, it is important to invest in the health and well-being of children and adolescents, nurturing their mental health, life skills, and resilience while protecting them from health-harming exposures. It is both a moral and developmental imperative to ensure a healthier and productive future for them.

Besides reducing premature mortality, morbidity too needs to be considered. While this is relevant for all NCDs, it is especially important for mental health disorders where morbidity contributes to a higher fraction of disease burden than premature mortality. It must also be recognized that different countries are currently in different stages of demographic transition, with different trajectories of the NCD epidemic. Demographically, many African countries will have proportionately more NCD-related deaths between 30–70 years as their populations age over the next two decades, while high and upper middle- income countries will experience the maximal burden of NCD in the eighth and ninth decades of life. Although the current SDG 3.4 goal is one-third reduction before 2030, we should aim for a minimum 50% reduction in NCD- related deaths and DALY across all ages below 70 years as a preferred goal for 2050.^3^ That revision is not only better aligned with the patterns of demographic and developmental transition in different countries but will also be more equitable across age and geography.

Since 2018, there has been a better understanding of the affordable, cost-effective, and feasible interventions (which have been termed by WHO as Best Buys) to prevent and treat NCD and mental health conditions, while providing the greatest return on investment. These include (a) policies and regulations targeting key risk factors such as tobacco use, harmful alcohol consumption, unhealthy diets, physical inactivity and air pollution; and (b) scalable, high-impact interventions for NCD and mental health, delivered through primary health care. As national and global level monitoring are key, the Global Monitoring Framework for NCDs needs to be revised to include updated mortality targets as well as measures for major morbidities associated with NCD and mental health.

All countries will need to intensify efforts to reduce NCD related disease burdens and risk factor exposure across the life course. They must ensure equal access to the benefits of these initiatives, while monitoring progress. Public policy in different sectors must be sensitive to, and aligned with, these goals. A wellbeing-economy approach should be applied to prioritize health as an investment rather than a cost, recognizing that economic policies directly influence the burden of NCD.

## NCD Must be the Prime Target for Achieving Healthy Life Expectancy in All Countries

As we chart the agenda for global health actions up to and beyond 2030, it must be clearly recognized that NCD are the leading global cause of avertable premature mortality and disability, presently contributing to 74% of all global deaths and 86% of all premature deaths in LMIC. Within the large cluster of NCD, cardiovascular diseases (CVD) are the largest contributors to premature mortality and disability, especially due to the high burden of coronary heart disease (CHD) and stroke. Over the past four decades, CVD has been the most responsive, amongst all NCD, to both population- based public health strategies and individually directed clinical interventions for CVD risk reduction. For these reasons, it is imperative that high priority be accorded by all countries to implement evidence-informed policy and health system interventions to prevent, identify, and treat CVD and related risk factors. The Lancet CIH3.0 identifies 15 health conditions that contribute to a large share of premature deaths in all countries, including hypertension and tobacco use. Global efforts should prioritize the health conditions that most affect low-income countries, while recognizing the countries’ own priorities. They must also amplify and utilize regional public health capacities. Progress will rest on national efforts to at least double domestic financial allocations to health while focusing investment on a limited package of services which are largely delivered through primary health care and community platforms.

## Effective Interventions Directed at NCD will Yield Collateral Benefits for Health and Sustainable Development

NCD also shares common causes and cross-cutting interactions with major established and emerging health challenges. As the recent COVID-19 pandemic and earlier influenza outbreaks in many countries showed, persons with pre-existing NCD or their risk factors have the highest risk of experiencing severe disease, hospitalization or death when infected. Intra-uterine and early childhood malnutrition are linked to adult NCD. At the same time, pregnancy-associated hypertension is a serious threat to maternal and child health. Hypertension can adversely affect the natural history of many infectious diseases. Gestational diabetes is rising in incidence alongside the obesity epidemic. Air pollutionand other environmental determinants of health are increasing the risk of NCD, ranging from CVD, diabetes and respiratory diseases to cancers and dementia. Alcohol abuse is culpable in many deaths and injuries related to traffic accidents, while also featuring as a major modifiable risk factor for NCD.

Even among environmental hazards to human health, upstream drivers of NCD are common. They involve atmospheric pollutants, deforestation, biodiversity loss, and industrial scale meat production for human consumption. Changes needed in food systems to combat NCD will also align with the objectives of a climate- smart and climate-resilient food system. Tobacco control not only has a profound impact on reducing the incidence and death rates of NCD but also curtails the environmental damage associated with tobacco production and consumption. Increased physical activity requires supportive changes in urban design and transport systems, which will also help countries to mitigate and adapt to climate change. Determined political action on CVD and other NCD will have many co-benefits for health and sustainable development. It should be a natural part of health preparedness and response in humanitarian settings.

## An Action Almanac that Extends Beyond 2030

Apart from increased financial allocations for NCD prevention and control programs, countries must also prioritize the development of a multi-layered, multi-skilled, digitally enabled health workforce which can collectively deliver the full range of needed health services. As populations age, health systems must enhance their capacity to deal with multi-morbidities. Community engagement and broad-ranging partnerships between government, civil society and industry will be needed to advance the NCD agenda through an all-of-society approach. Implementation research needs to be scaled up to effectively utilize the impactful interventions we possess to benefit all populations. We must strive to make health products and technological innovations– including the ethical use of artificial intelligence, machine learning, and large language models– cheaper, more robust, and effective across a wide range of NCD to drive global health gains. Together, established and new partnerships can advance coordinated clinical trial networks and increase funding for developing multi-use platforms, such as mRNA therapeutics. Developments in artificial intelligence have the potential to accelerate discovery times, while regulatory harmonization will enable a reduction in delays due to misaligned national licensing processes.

## Summary

CVD, mostly manifest in the form of CAD and stroke, is the leading contributor to adult mortality and is the foremost cause of premature deaths in the most populous parts of the world, represented by LMIC. As the world rededicates its commitment to overcome the health and developmental threats posed by NCD, effective interventions directed at CVD must receive the highest priority in multi-sectoral policies and health system practices. Support must be tailored to country priorities and capacities to encourage and support the development of national systems, as recently underscored in the Lusaka agenda^4^ with its roadmap for strengthening global health initiatives (GHI) and domestic financing for health in support of universal health coverage (UHC).

## WHF Commitment and Call to Action

While declaring its unbounded commitment to the achievement of a long and healthy life expectancy for all populations, as reflected in the SDG, the World Heart Federation calls upon all nations to effectively utilize the knowledge and experience gathered in reducing the burdens of CVD to achieve the overall NCD targets, while reaping many co-benefits for sustainable development.

## Footnotes

In 2021, cardiovascular diseases accounted for at least 19 million deaths, equivalent to 28 per cent of global mortality. In 2019, hypertension affected approximately one-third of adults globally – double the number reported in 1990, with only one in five having their blood pressure under control. By 2022, more than 800 million adults were living with diabetes– a four-fold increase since 1990. In 2022, more than one billion people were estimated to be living with obesity. In 2021, close to one billion people were also estimated to be living with a mental disorder, including 8% of the world’s young children (aged 5–9 years) and 14% of the world’s adolescents (aged 10–19 years). In 2021, NCDs and mental health conditions together accounted for four out of every five years lived with disability.

World Health Organization. Billions left behind on the path to universal health coverage [Internet]. 2023 Sep 18 [cited 2025 Aug 25]. Available from: https://www.who.int/news/item/18–09–2023-billions-left-behind-on-the-path-to-universal-health-coverage.

Global Health 2050: the path to halving premature death by mid-century [Internet]. Lancet Commission on Investing in Health (CIH). GlobalHealth2050.org; 2024 Oct 14 [cited 2025 Aug 25]. Available from: https://globalhealth2050.org/files/2024/10/CIH-report-brief_final.pdf.

The Lusaka Agenda: Conclusions of the Future of Global Health Initiatives Process [Internet]. Future of Global Health Initiatives; 2023 Dec 12 [cited 2025 Aug 25]. Available from: https://d2nhv1us8wflpq.cloudfront.net/prod/uploads/2023/12/Lusaka-Agenda.pdf.

